# Interference of *Aspergillus fumigatus* with the immune response

**DOI:** 10.1007/s00281-014-0465-1

**Published:** 2014-11-18

**Authors:** Thorsten Heinekamp, Hella Schmidt, Katrin Lapp, Vera Pähtz, Iordana Shopova, Nora Köster-Eiserfunke, Thomas Krüger, Olaf Kniemeyer, Axel A. Brakhage

**Affiliations:** 1 Department of Molecular and Applied Microbiology, Leibniz Institute for Natural Product Research and Infection Biology (HKI), Jena, Germany; 2 Integrated Research Treatment Center, Center for Sepsis Control and Care (CSCC), University Hospital Jena, Jena, Germany; 3 Department of Microbiology and Molecular Biology, Institute of Microbiology, Friedrich Schiller University Jena, Adolf-Reichwein-Straße 23, 07745 Jena, Germany

**Keywords:** *Aspergillus fumigatus*, Immune evasion, Phagocytes, Epithelial cells, Neutrophil extracellular traps (NETs), (immuno-) proteomics, Complement

## Abstract

*Aspergillus fumigatus* is a saprotrophic filamentous fungus and also the most prevalent airborne fungal pathogen of humans. Depending on the host’s immune status, the variety of diseases caused by *A. fumigatus* ranges from allergies in immunocompetent hosts to life-threatening invasive infections in patients with impaired immunity. In contrast to the majority of other *Aspergillus* species, which are in most cases nonpathogenic, *A. fumigatus* features an armory of virulence determinants to establish an infection. For example, *A. fumigatus* is able to evade the human complement system by binding or degrading complement regulators. Furthermore, the fungus interferes with lung epithelial cells, alveolar macrophages, and neutrophil granulocytes to prevent killing by these immune cells. This chapter summarizes the different strategies of *A. fumigatus* to manipulate the immune response. We also discuss the potential impact of recent advances in immunoproteomics to improve diagnosis and therapy of an *A. fumigatus* infection.

## Introduction

Fungal pathogens cause a wide range of diseases ranging from allergies and superficial infections to life-threatening invasive mycoses. Often, the outcome of a fungal infection depends on the immune status of the host organism. In particular, individuals with a compromised immune system represent a group at high risk to develop fatal fungal infection. The continuous progress in intensive care, e.g., in chemotherapy and organ or bone marrow transplantation, contributes to the steadily increasing number of patients with impaired immune status [1].

The genus *Aspergillus* comprises more than 250 species, including on the one hand “good guys” that are industrially used for production of pharmaceuticals, beverages, and food additives; but on the other hand also, several “bad guys” that call for toxin-based crop spoilage or being the causative agent of severe fungal infections. Among the latter group, *Aspergillus fumigatus* is the number one airborne fungal pathogen of humans. To date, neither reliable diagnostic tools nor effective treatment options are available resulting in unacceptable high mortality rates of patients suffering from invasive fungal infections [2]. Therefore, the identification of new diagnostic markers and the development of novel therapeutics for specific intervention are of great importance. Especially, the characterization of the pathogen’s strategies to defend against attacks of host immune cells is interesting to understand pathogenicity and is important for the identification of potential therapeutic targets.

**Fig. 1:**
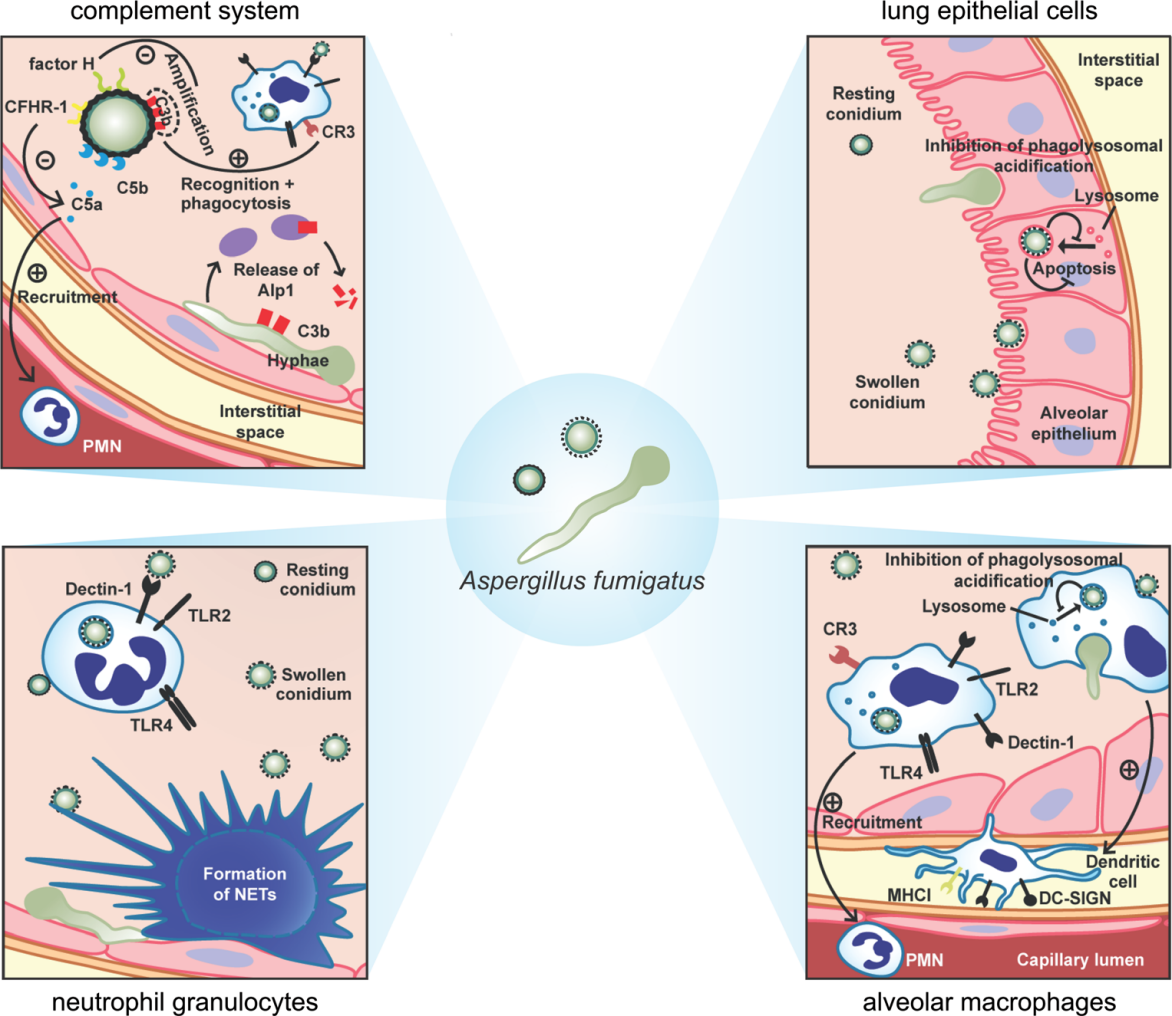
Schematic depiction of the interaction between *A. fumigatus* and the immune system of the host (for details see text)

**Fig. 2:**
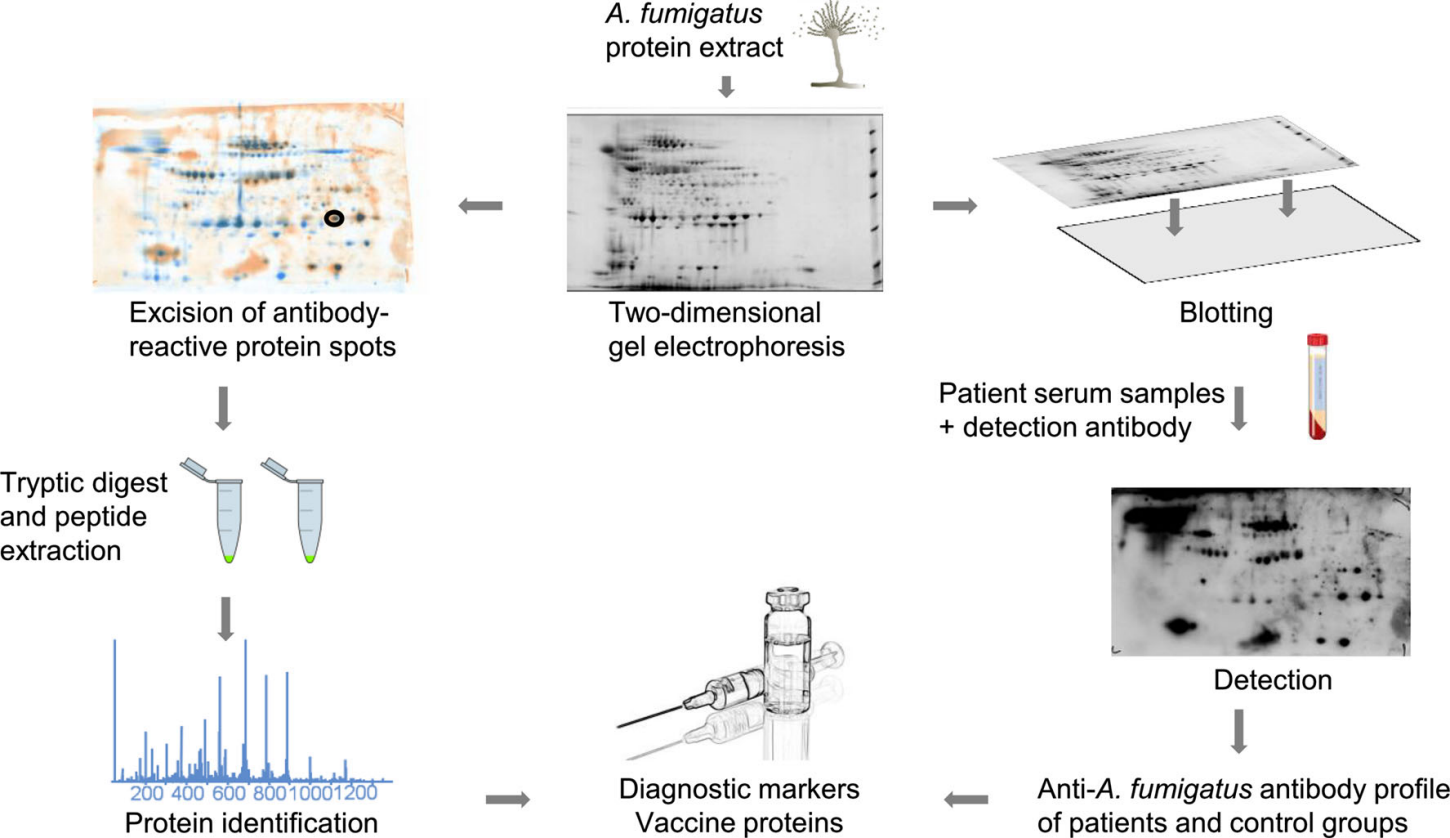
Identification of immunoreactive proteins of *A. fumigatus* by immunoblot analysis of 2D-electrophoresis maps with patient sera

This article focuses on the interaction of *A. fumigatus* with components of the human immune system. In detail, we discuss the various strategies of this fungus to interfere with lung epithelial cells and phagocytes such as macrophages and neutrophils, and we illustrate how *A. fumigatus* evades the human complement (Fig. 1). Finally, we will discuss recent advances in immunoproteomics and their impact on target identification and improvement of diagnosis (Fig. 2).

### Interaction of *A. fumigatus* with lung epithelial cells

Conidia of *A. fumigatus* are comparatively small with a diameter of only 2–3 μm. They are propagated easily through the air and enter the human host via the airway, where they infect the lung tissue and intrude to the lower respiratory system [3]. Epithelial cells of the lung therefore represent the first contact barrier where *A. fumigatus* interacts with host cells. The alveolus is lined by alveolar epithelial type I and type II cells and especially type II pneumocytes maintaining the alveolar space are confronted to inhaled conidia. In contrast to macrophages or neutrophils, pulmonary epithelial cells represent nonprofessional phagocytes. Upon contact, conidia were shown to strongly adhere to type II pneumocytes of the A549 cell line, which then start to engulf the fungus. Endocytosed conidia are able to survive and reside inside A549 cells [4, 5].

According to the fact that manipulation of host cell apoptosis is an important strategy of many pathogens to establish an infection, it was found that *A. fumigatus* inhibits apoptosis in different epithelial cell types [6]. Only recently, it was shown that the fungal 1,8-dihydroxynaphthalene (DHN)-melanin is responsible for this effect on the epithelial apoptosis process [7]. DHN-melanin is also essential to prevent phagolysosomal acidification in alveolar epithelial cells to survive intracellularly. A current hypothesis is that since phagocytic activity of epithelial cells is rather low, some conidia might persist within these cells and thereby represent the infectious reservoir after impairment of the host’s immune system [7].

Interestingly, the mechanisms by which *A. fumigatus* interfere with nonprofessional phagocytes is largely similar to professional phagocytic cells, i.e., macrophages and neutrophils, as described in detail in the following.

### Interaction of *A. fumigatus* with alveolar macrophages

Invading conidia in the lung tissue encounter the resident leucocytes which constitute the first line of the host’s immune defense. Alveolar macrophages are homed just beneath the alveolar surfactant film and represent 90 % of the resident leucocytes in the lung [8]. They are credited with a major contribution to the initial immune response against *Aspergillus* infections besides neutrophil granulocytes. Alveolar macrophages originate from immigrating blood monocytes or from precursor cells residing in the lung [8]. Patients with reduced numbers of those phagocytes, e.g., due to lymphatic or leukemic malignomes or after stem cell or solid organ transplantation, have increased susceptibility towards aspergillosis [2, 9]. The recognition of conidia by alveolar macrophages leads to phagocytosis and induces the expression of inflammatory chemokines and cytokines such as TNF*-α*, IL-12, IFN-γ, IL-18, IL-6, IL-1β, MIP-1, MIP-1α, MIP-2, G-CSF, and GM-CSF [10–12]. They recruit further professional phagocytes such as circulating macrophages, neutrophil granulocytes, and dendritic cells (DCs) to the site of infection. The interaction of *A. fumigatus* with neutrophils is described in more detail below. Dendritic cells are able to phagocytose conidia and even hyphae, and their function in antigen presentation via the major histocompatibility complex class I (MHC I) is important to initiate the adaptive branch of the immune system by the activation of T cell and antibody responses [13, 14].

The fungal surface is composed of α- and β-glucans, chitins, galactomannans, and other polysaccharides. In particular, β-1,3-glucans and galactomannans are highly immunogenic molecules and prominent pathogen-associated patterns (PAMPs) of fungi [15, 16]. However, the rodlet layer composed by the hydrophobic RodA protein and the pigment DHN-melanin on the surface of resting *A. fumigatus* conidia render the conidia largely inert towards recognition by the immune system [17]. Swelling and germination of conidia are accompanied by a subsequent loss of the protective melanin and hydrophobin layer and thereby lead to an increasing exposure of immunological surface components and enhanced phagocytosis [16, 18]. Pigmentless strains, due to deletion or mutation of the *pksP* gene encoding the polyketide synthase essential for melanin biosynthesis, display a smooth surface structure and enhanced phagocytosis during co-incubation with macrophages [19–21]. DHN-melanin has been shown to be important for correct cell wall assembly and ascribed a function in the defense against the host’s immune response [22, 23]. Remarkably, a lack of DHN-melanin is accompanied with attenuation of virulence [19, 24].

Different pathogen recognition receptors (PRRs), which are expressed by cells of the innate immune system, are responsible for the detection of specific PAMPs [25]. The DC-specific intracellular adhesion molecule 3-grabbing nonintegrin (DC-SIGN), which is mainly expressed on DCs, binds fungal galactomannans also on macrophages and initiates phagocytosis [26]. Toll-like receptors (TLRs) 2 and 4 have been attributed with immune recognition and modulation of the immune response in *Aspergillus* infections [27–29], although their actual function in invasive aspergillosis has not been rigorously elucidated yet. TLR2 was ascribed to sense chitin on the fungal surface, and TLR4 presumably detects α-glucans [30, 31]. A contribution of TLR9 to spore detection is discussed [32]. The main route of fungal recognition is the specific binding of the C-type lectin dectin-1 to β-1,3-glucans which are exposed on the fungal surface [11]. Dectin-1 is expressed on macrophages, monocytes, neutrophils, DCs, and a subset of T cells [33].

After pathogen recognition, phagocytosis is induced. Within the phagocyte, the conidium-containing phagosome fuses with lysosomes to form the phagolysosomes. Killing of the pathogen is observed starting around 6 h after ingestion [34]. Swelling of the conidium and acidification of the phagolysosome is prerequisite for efficient elimination [35]. During the fusion events with lysosomes, hydrolytic enzymes are exchanged. They comprise proteases, most importantly the cathepsins D, B, H, L, and S [36]. Further enzymes are hydrolases such as β-hexosaminidase and β-glucoronidase, lipases, and DNases [37]. Upon acidification of the compartment, these hydrolytic enzymes gain their catalytic activity towards the ingested particle.

A requirement for the biocidal property of the phagolysosome is the acidification of the compartment. The vacuolar ATPase (vATPase), a multiprotein membrane complex, has a major contribution in lowering the intracompartmental pH from pH >6 to pH <4.5 by pumping H^+^ ions across the membrane in an ATP-dependent reaction [35]. Treatment of macrophages with the vATPase inhibitor bafilomycin A resulted in a complete abolishment of acidification and confirmed the major role of the vATPase in the phagolysosomal acidification [38]. Conidia of the *A. fumigatus* wild type are localized to a much lower rate in such acidified compartments. Thywissen et al. [39] found the conidial pigment DHN-melanin to be a crucial factor for the inhibition of phagolysosomal acidification. In assays to investigate phagocytosis and intracellular processing of wild-type conidia compared to conidia of the pigmentless *pksP* mutant, wild-type conidia revealed retarded uptake by macrophages compared to mutant conidia. The intracellular phagolysosomal maturation process, especially fusion events with endosomes and lysosomes, was reported to be similar for both strains. By contrast, a striking difference in the acidification of the phagolysosome was observed. Whereas phagolysosomes that contained *pksP* mutant conidia acidified to pH below 4.5, the wild-type conidia containing phagolysosomes showed no reduction of pH. Melanin ghosts showed the same effect of reduced acidification, emphasizing the role of DHN-melanin in this mechanism [24, 39]. Other melanins such as DOPA melanin were not able to inhibit phagolysosomal acidification [40]. A contribution of the hydrophobin layer could be excluded, because conidia of a ∆*rodA* mutant strain did not inhibit acidification of the phagolysosome [17, 24, 39]. Furthermore, DHN-melanin deficiency did not interfere with the formation of rodlet structures on the conidial surface [39].

Melanized conidia of *A. fumigatus* were shown to inhibit apoptosis by activation of PI 3-kinase/Akt signaling pathway [41]. The internalization of melanized conidia prevented macrophages from cell death when apoptosis was induced via the intrinsic pathway with staurosporine or via the extrinsic pathway with the Fas ligand [41, 42]. Opposed to nonpigmented conidia or hyphae, a sustained PI 3-kinase/Akt signaling was observed in macrophages, epithelial cells, and pneumocytes that resulted in activation of the protein kinase B (PKB) and sustained levels of antiapoptotic proteins of the Bcl family such as Bcl-2 and Mcl-1, reduced cytochrome c release from mitochondria, and consequently reduced apoptosis promoting activation of effector caspases 3, 6, and 7. PI 3-kinase/Akt signaling was demonstrated to further enhance activation of FoxO and NF_k_B that initiates gene expression and production of inflammatory cytokines [6, 41–43]. The effect was found to be unique for different melanins, including DHN-melanin, DHN-melanin precursors, and DOPA melanin. Volling and co-workers proposed a two-step strategy comprising first the inhibition of acidification of the phagolysosome which then, secondly, resulted in inhibition of apoptosis. The apoptosis inhibition was further attributed to the reactive oxygen intermediates (ROI) quenching effect of melanins: Enhanced ROI signaling usually promotes cytochrome c release but is counteracted by the presence of the pigment on internalized conidia [41]. In contrast, germlings and hyphae of *A. fumigatus* induce apoptosis via the release of the toxin gliotoxin in a dectin-1 and TLR2-dependent manner to evade immune responses [44, 45]. It was hypothesized that inhibition of apoptosis by *A. fumigatus* conidia creates a protective intracellular niche for the fungus to evade fungicidal effects of immune cells and to escape the disruption of infected cells by cytotoxic T cells as well as a shuttle for transport to lymph nodes and spleen [24, 46].

### Interaction of *A. fumigatus* with neutrophil granulocytes

Beside macrophages, neutrophil granulocytes are important effector cells of the innate immunity. They represent the most abundant immune cells and belong to the first line of defense against bacterial and fungal infections. Each day, around 5–10 × 10^10^ neutrophils are produced in the bone marrow [47]. After full differentiation, they are released into the bloodstream where they have a limited lifespan of about 6–8 h. However, without external stimuli, neutrophils can circulate for up to 5 days outside the bone marrow [48]. In the event of infection, a large number of neutrophils is recruited to the affected tissue in response to cytokines such as IL-8, IFN-γ, C5a, and leukotriene B4 [49]. Neutrophils are the key effector cells in the immune response against *A. fumigatus*. Accordingly, neutropenic patients face the highest risk to develop an invasive aspergillosis [50]. Depletion of neutrophils in mice infected with *A. fumigatus* conidia induced high mortality rates and hyphae-induced lesions in the lung. In contrast, infected mice with depletion of macrophages were still able to prevent conidial germination resulting in 100 % survival [51].

Neutrophils recognize *A. fumigatus* at least via dectin-1 and TLRs 2 and 4 [52–54] and possess different mechanisms to kill the fungus. As professional phagocytes, they ingest conidia and germlings very rapidly to kill them by a respiratory burst and degranulation [55, 56]. Neutrophils contain three different types of granules. Primary or azurophilic granules contain myeloperoxidase (MPO), lysozyme, and antimicrobial proteins like cathepsin G, elastase, and proteinase 3. Secondary or specific granules hold lactoferrin, lysozyme, lipocalin, and membrane proteins like flavochrome b_558_. Tertiary granules contain gelatinase [57, 58]. Upon activation, neutrophils secrete cytokines and chemokines, e.g., CXCL1/2/3, CCL2/3/4, and IL-8 to attract further immune cells [52, 59]. Neutrophils attach to hyphae that are too big to be phagocytosed. By means of degranulation and oxidative and non-oxidative killing mechanisms, fungal hyphae are damaged [60]. Oxidative killing is depending on the assembly of the NADPH oxidase. This enzyme complex produces superoxide anions that are further converted to toxic compounds like H_2_O_2_, hydroxyl anions, and hypochlorous acid [61]. Patients suffering from chronic granulomatous deficiency (CGD), an inherited disorder caused by defects in the NADPH oxidase subunit gp91^*phox*^, are at high risk for invasive *Aspergillus* infections. Neutrophils from gp91^*phox*^-deficient mice or CGD patients have impaired fungicidal activity in vitro [55, 62]. Co-incubation of *A. fumigatus* hyphae with human neutrophils led to upregulation of genes encoding catalases and cytochrome c peroxidase [63]. Although previous studies hypothesized that ROI might be involved in killing of *A. fumigatus*, ROI detoxification mutants of *A. fumigatus* revealed no difference in virulence in mouse infection models when compared to the *A. fumigatus* wild type [64–66]. Therefore, it seems highly unlikely that ROI play an essential role in killing of this fungal pathogen.

Neutrophils can also produce reactive nitrogen intermediates (RNI) against microorganisms [67]. RNI can easily diffuse through membranes, nitrosylate proteins, and damage membranes and DNA. RNI can react with ROI to form toxic products like peroxynitrite. RNI also function as signal molecules in the immune response [67]. *A. fumigatus* employs two systems to detoxify RNI: flavohemoglobins (FhpA and FhpB) and the S-nitrosoglutathione reductase GnoA [68]. However, virulence in a murine model of pulmonary aspergillosis was not dependent on the ability of the fungus to counteract RNI produced by host immune cells [68].

Neutrophils can also use non-oxidative mechanisms to fight against pathogens. One is the discharge of the granule content, designated as degranulation. Defensins exert fungistatic activity and can kill *A. fumigatus* extracellularly [69, 70]. Zarember et al. [56] showed that lactoferrin can inhibit fungal growth by sequestering free iron ions. Furthermore, serine proteases like the PMN elastase (ELA) contribute to microbial killing [71, 72]. In line, ELA knockout (ELANE) mice exhibited a higher fungal burden indicating that ELA is involved in fungal clearance in vivo. However, in a murine model for invasive aspergillosis, ELANE mice showed survival rates like wild-type mice [73], indicating that the meaning of ELA, if there is any, is more complex.

A decade ago, Brinkmann et al. [74] described another extracellular killing mechanisms of neutrophils, the formation of neutrophil extracellular traps (NETs). It was shown in vitro and in mice that neutrophils produce NETs upon contact with *A. fumigatus* [75]. NETs consist of DNA fibers decorated with histones and antimicrobial proteins. NETosis is a specific form of cell death, at which the cell content is mixed and released into the surrounding. ROI produced by the NADPH oxidase complex are required for NETosis, which can also be induced exogenously by phorbol-12-myristate-13-acetate (PMA), LPS, and IL-8 [76]. In addition to histones, NETs mainly contain the proteins elastase, lactoferrin, cathepsin G, calprotectin, and MPO [77]. The decondensation of chromatin is regulated by neutrophil elastase and MPO [71]. Also, the Raf-MEK-ERK-pathway plays a role in the formation of NETs [78]. Obviously, NETs attach to and attack the pathogen by antimicrobial proteins. Calprotectin has a major antifungal effect, but also defensins, cathelicidin LL37, and histones show antifungal activity [79–81]. Pentraxin 3 (PTX3) is a soluble pattern recognition receptor produced by specific cells but also stored in granula in neutrophils. It is released also during formation of NETs and exhibits anti-*Aspergillus* activity [79, 82]. Exogenous addition of PTX3 early in infection restored antifungal resistance and restrained the inflammatory response to *A. fumigatus* [83]. Also, genetic deficiency of PTX3 affects the antifungal capacity of neutrophils and might contribute to the risk of invasive aspergillosis in hematopoietic stem cell transplantation patients [84]. Although NETs effectively kill several bacteria and pathogenic fungi such as *C. albicans* [74, 80, 81], they only act fungistatically against *A. fumigatus* [75, 85].

### *A. fumigatus* evades the human complement system

The human complement system comprises approximately 30 serum-derived or membrane-associated proteins to exert its manifold effects on host-pathogen interactions and inflammatory events [86]. These effects aim at the maintenance of tissue homeostasis, resolution of inflammation, and clearance of pathogens, apoptotic cells, or debris. Moreover, the complement system provides a platform for the cross-talk between innate and humoral circuits via the interaction of complement activation products and surface receptors resulting in governing both T and B cell responses [87]. The non-redundant role of the complement system in the onset of invasive aspergillosis has been well documented in DBA/2N mouse models [88]. Complement system deficiencies correlate with a higher mortality rates in C5 knockout mice challenged with *A. fumigatus* [89, 90].

Complement activation can be triggered by three distinct pathways: the classical, the alternative, and the lectin pathway, all leading to proteolytic cleavage of the central C3 complement factor by C3 convertases and activation of the terminal pathway. Terminal pathway products include C3a and C3b that lead to the formation of terminal complement complex (TCC) and membrane attack complex (MAC) [87].

The complement machinery is induced by both *Aspergillus* conidia and hyphae. Resting conidia mainly activate complement by the alternative pathway. Conidial germination and hyphal formation, processes accompanied by changes of the cell wall composition, and exposure of surface α- and β-glucans shift complement activation to the classical pathway. Complement activation by all three forms of *A. fumigatus* features specific kinetics with slowest initiation by resting conidia [91].

Opsonization of pathogens by C3 complement fragments is a key strategy in complement-mediated clearance. Complement opsonization represents a chemotactic compass for immune cells and enhances phagocytosis, ROI production, and killing by alveolar macrophages, monocytes, and neutrophils [87]. Opsonization strongly depends on the availability of putative docking sites for C3 fragments on the fungal surface. A strong hint supporting this hypothesis was provided by two findings. Firstly, DHN-melanin serves as an efficient camouflage tool that reduces the exposure of surface antigens for C3 binding thus masking the fungal spores from efficient complement opsonization. DHN-melanin-deficient conidia bind more C3 fragments on their surface and exhibit reduced virulence in mice [19, 92]. These findings, together with reports of DHN-melanin as a scavenger of ROI and as an inhibitor of phagolysosome acidification in phagocytes, portrait DHN-melanin as a primary tool for *A. fumigatus* to hijack immune surveillance [46]. Secondly, complement deposition has been shown to be markedly different between virulent and non-virulent Aspergilli with *A. fumigatus* and *A. flavus* binding significantly less C3 in comparison to *A. glaucus* and *A. nidulans* [90].

The glucan surface cell wall carbohydrate structures can bind mannose-binding lectin (MBL), facilitate activation of the lectin pathway, and lead to C4 deposition in a concentration-dependent manner. MBL can then back up C3 proteolytic cleavage via C2 bypass mechanism disengaging the C3 convertase and thus initiating the alternative pathway. Such a scenario is not a specific hallmark of *A. fumigatus* alone but is also valid for *A. flavus*, *A. niger*, and *A. terreus* [93]. Crosdale and colleagues [94] highlighted the crucial role of serum MBL levels in the immune clearance of Aspergilli by investigating the correlation between the presence of mutations in the MBL gene promoter region that downregulated serum levels of MBL and the severity of chronic necrotizing pulmonary aspergillosis [94, 95].

*Aspergillus*-driven complement activation can also occur via the opsonization by PTX3 receptor that interacts either with ficolin-2 [96] via the lectin pathway or associates with C1q from the classical pathway [97]. In agreement with these findings, a recent report linked a single nucleotide gene polymorphism in PTX3 in homozygous haplotype donor individuals to an increased risk of invasive aspergillosis in stem cell recipients of such donor cells [84].

The fine tuning of the regulation of the complement machinery is achieved by the action of complement inhibitors. *A. fumigatus* sabotages complement activities by acquisition of complement inhibitors such as factor H (FH), factor H-related protein FHL-1, a splicing product of the FH gene, and C4 binding protein (C4bp). Factor H-binding sites were pinpointed to the N-terminal short consensus repeats (SCRs) 1 to 7 and one in the C-terminal SCR 20. FH and C4bp are soluble proteins promoting cleavage of C4b and C3b and accelerating disintegration of assembled C3 convertase. As a functional consequence, complement system is downregulated [98]. Furthermore, *A. fumigatus* produces complement inhibitor (CI) with yet unresolved structure that interferes with the alternative pathway and C3b-driven phagocytosis and killing [99].

*A. fumigatus* evades the complement also via secretion of extracellular proteases. For example, Alp1, a serine protease shown to degrade collagen, fibrinogen, and other extracellular matrix proteins, also targets complement components such as C1q, C3, C4, C5, MBL, and factor D [100, 101].

Taken together, *A. fumigatus* has developed a wide variety of armory to combat complement system activities. Unraveling the tools of such armory contributes to the elucidation of pathogenesis mechanisms and the development of therapeutic approaches for invasive aspergillosis by interference with the human complement system.

### Immunoproteomics to identify *A. fumigatus* antigens

As described in the previous sections, interaction of *A. fumigatus* with the immune system takes place at several levels during infection of the human host. Next to biochemical and phenotypic single factor analysis and the application of other *omics* methods like transcriptomics, (immuno-) proteome approaches have been applied to investigate *Aspergillu*s proteins that are likely communicating with host cells and effector proteins of the innate and adaptive immune system during infection. In general, proteomic techniques involve a protein cleanup and the reduction of the sample complexity by single or multi-dimensional electrophoretic and/or chromatographic separation procedures prior or subsequent to the enzymatic protein digestion [102]. The resulting peptide sequences are commonly measured by high-resolution tandem mass spectrometry and finally matched and statistically evaluated by sophisticated protein database search algorithms such as Mascot or Sequest [102].

Besides investigation of the dynamics of the *A. fumigatus* (wild-type or mutant) proteome, when cultivated under different conditions, e.g., upon confrontation with particular stresses [65, 103–106], *Aspergillus* cell wall and cell membrane subproteomes are of especial interest in the context of *A. fumigatus* pathogenicity. The fungal cell wall and the cytoplasmic membrane have a crucial impact on the virulence of the pathogen by constituting the first barriers interacting with the environment and consequently with the host’s immune defense [107]. Although proteins are only minor components of a fungal cell wall which is mainly composed of polysaccharides, they can play a major role in the interaction with the host, especially as antigens/allergens, adhesins, enzymes, or immunomodulators. In *A. fumigatus*, several conidial surface proteins such as the surface layer protein (hydrophobin) rodlet A, the aspartic protease PEP2, a putative disulfide isomerase, or an extracellular lipase, have been identified upon β-1,3-glucanase treatment of dormant conidia [108]. The presence of an extracellular lipase on the conidial surface may induce cell damage and adherence of the spores within the bronchoalveolar tract during inhalation [108]. In addition, proteins localized within the cytoplasmic lipid bilayer are also crucial for the interaction of the fungus with the environment and, in particular, with the host’s immune system. Ouyang et al. [109] analyzed total membrane preparations of *A. fumigatus* by SDS-PAGE separation, in-gel digestion, and a subsequent 2D-LC-MS/MS analysis. Thereby, the authors identified 530 membrane-associated proteins of which 17 were integral membrane proteins involved in N-, O-glycosylation, or glycophosphatidylinositol (GPI) anchor biosynthesis. For identification of GPI-anchored proteins, Bruneau et al. [110] released these proteins in a soluble form from the membrane fraction prior to proteome analysis by the combined action of the detergent n-octylglucoside and an endogenous phospholipase C activity.

In addition to analyzing solitary cultures, it has recently become attractive to directly investigate the interplay of *Aspergillus* with selected host cells by studying the changes in the individual proteomes and the interactome of co-cultures by proteomic (and transcriptomic) procedures [63, 111–113]. These strategies are especially suitable to dissect the interrelationships between host cells and the pathogen during infection. A study focusing on the proteome of human umbilical vein endothelial cells (HUVECs) upon co-incubation with *A. fumigatus* was recently performed [114]. By applying a mass spectrometry label-free proteomic approach, the molecular mechanism by which *A. fumigatus* can activate human HUVECs during blood vessel invasion was investigated. Angioinvasion is a key feature of invasive pulmonary aspergillosis (IPA). Endothelial cells act as physiological barriers that facilitate leukocyte migration and local immune response against microbial pathogens by secretion of cytokines/chemokines and other signal molecules. Vascular invasion of *A. fumigatus* leads to an activated prothrombic phenotype of HUVECs by which leukocyte migration, and an effective immune response is inhibited. A total of 89 proteins were differentially regulated during interaction of HUVECs with *A. fumigatus* germlings, i.e., 57 proteins were downregulated and 32 were upregulated. Another 409 proteins have been detected that were exclusive to one experimental condition (treatment or control). The group of upregulated proteins or proteins that have been exclusively identified in the interaction of HUVECs with *A. fumigatus* included particularly proteins with proangiogenic properties, namely, intercellular adhesion molecule-1 (ICAM-1), hepatocyte growth factor (HGF), fibroblast growth factor (FGF), activated leukocyte cell adhesion molecule (ALCAM), and basignin. As a consequence, it has been suggested that the vascular invasion by *A. fumigatus* activates multiple proteins that are involved in angiogenesis [114].

Another important focus in the field of immunoproteomics is the identification of *Aspergillus* proteins that serve as allergens and antigens in different *Aspergillus*-derived diseases by provoking a cellular and humoral immune response. Sensitization is one undesirable effect of the immune system that might arise when in contact with *Aspergillus* species (mostly *A. fumigatus*) due to prolonged inhalation and/or colonization of the lung. In particular, individuals suffering from cystic fibrosis or asthma are prone to develop an allergic reaction to *Aspergillus*, which can manifest itself as allergic pulmonary aspergillosis (ABPA) and exacerbates the health status of these patients [115]. ABPA is proposed to arise as a consequence of an inflammatory response to *Aspergillus* allergens, and *Aspergillus* causes bronchial epithelial cell damage that triggers a Th2 hypersensitivity immune response. Therefore, the clinical picture is generally accompanied with an elevated total IgE level and *Aspergillus*-specific elevated IgE and IgG levels [115]. Such anti-*Aspergillus* antibody profiles of ABPA patients (and of *A. fumigatus* sensitized asthmatics) have been analyzed in a number of immunoproteomic studies [116–119]. Gautam et al. [116], for example, studied the IgE immunoreactivity of *A. fumigatus* proteins by 2D gel electrophoresis followed by immunoblotting of 3-week old culture filtrate proteins with sera from *A. fumigatus*-sensitized asthmatics and ABPA patients. The authors identified five known and 11 novel antigens by MALDI-TOF mass spectrometry, including an extracellular arabinase, a chitosanase, and a catalase. Based on an enzyme-linked immunosorbent assay (ELISA), Glaser et al. (2009) showed that 94 % of ABPA patients (*n* = 64) and 46 % of *A. fumigatus*-sensitized asthmatics (*n* = 24) had Asp f 34-specific serum IgE [120]. Next to a better understanding of the disease, identified allergens can be used as recombinant proteins in novel ABPA-diagnosing assays that are based on the identification of anti-*Aspergillus* IgE and/or IgG antibodies in sera of patients. Interestingly, for ABPA, so far, only five recombinant proteins (rAsp f1–f4 and f6) of more than 20 known antigens/allergens are commercially available to proof an allergic immune response against *A. fumigatus* [121].

Such immunoproteomic analyses of *Aspergillus* proteins with respect to their interaction with patients’ antibodies have been also extended to cohorts of patients suffering from invasive forms of *Aspergillus* infections, like IPA. Although the highest risk group of patients suffering from invasive aspergillosis (IA) is strongly immunocompromised, there is a rising incidence of IA in non-immunocompromised but critically ill patients (up to 5.8 %). Shi et al. (2012) tested the immunoreactivity of 2D gel electrophoresis-separated extracellular proteins of *A. fumigatus* with sera from such patients with proven IA [122]. The authors identified 17 different antigens by application of MALDI-TOF mass spectrometry. The most intense immunoreactivity could be assigned to the secretory gliotoxin oxidase GliT (TR). Antibodies specific to TR have been proposed as a potential biomarker for the serologic diagnosis of IA in non-neutropenic patients that exhibit low serum galactomannan sensitivity [123].

Aside from detection of relevant *Aspergillus* proteins that have to be expressed during infection to elicit an immune response and their potential as diagnostic markers, the identification of vaccine candidates is an important objective in immunoproteomic approaches [124]. For identification of protective antibodies, Asif et al. (2010), for example, investigated two infected rabbits developing a protective immune response against invasive *Aspergillosis* by application of a 2D gel electrophoresis immunoblotting approach that has been combined with an identification of the antigenic proteome by LC-MS/MS determination [125]. In total, the authors identified 59 antigens, including proteins related to glycolysis and other primary metabolic pathways, oxidative stress response, and protein folding (heat shock proteins) as potential vaccine candidates.

## Conclusions

*A. fumigatus* has developed a number of immune evasion mechanisms which interfere at the different levels of the infection process with the response of the human host. These include recognition of conidia, modulation of phagocytosis, intracellular processing, NET formation, and complement activation. New techniques and recent advances to analyze changes on the molecular level in both the fungal pathogen and host cells provide exciting possibilities to pin down essential steps in host pathogen interaction. For example, next generation sequencing allows expeditious acquisition of genome information of a multitude of *A. fumigatus* clinical and environmental isolates. RNAseq and sophisticated LC-MS analyses enable monitoring interaction-induced changes on the transcript level including alternative splicing, RNA editing, and protein modifications on both sides, the pathogen and the host. Last and not the least, automated analysis of digitalized and processed images or movies of the interaction of the pathogen with host cells was recently established [126]. Combined in a systems biology approach [127, 128], all these data will allow to model the interaction of fungal pathogens with different immune cells and to identify regulatory circuits of this interaction with the overall aim to improve diagnosis and to identify novel targets for the development of tailor-made antifungal drugs.
